# Perform tumor-specific survival analysis for Merkel cell carcinoma patients undergoing surgical resection based on the SEER database by constructing a nomogram chart

**DOI:** 10.1515/med-2024-1103

**Published:** 2025-03-21

**Authors:** Jingxuan Zhou, Hai Yu, Xichun Xia, Yanan Chen, Wai-kit Ming, Yuzhen Jiang, Yau Sun Lak, Chongchong Ip, Chaodi Huang, Qiqi Zhao, Suzheng Zheng, Liming Xia, Xinkai Zheng, Shi Wu, Jun Lyu, Liehua Deng

**Affiliations:** Department of Dermatology, The First Affiliated Hospital of Jinan University & Jinan University Institute of Dermatology, Guangzhou, China; Institute of Biomedical Transformation, Jinan University, Guangzhou, China; Institute of Dermatology and Venereal Diseases, Dermatology Hospital, Southern Medical University, Guangzhou, 510091, China; Department of Infectious Diseases and Public Health, Jockey Club College of Veterinary Medicine and Life Sciences, City University of Hong Kong, Hong Kong, China; Department of Ophthalmology, Royal Free Hospital & University College London, London, United Kingdom; Department of Dermatology, Centro de Hospitalar Conde de Januario, Macau, China; Department of Dermatology, University Hospital Macau, Macau, China; Department of Dermatology, The Fifth Affiliated Hospital of Jinan University, Heyuan, China; Guangzhou Jnumeso Bio-technology Co., Ltd., Guangzhou, China; Department of Clinical Research, The First Affiliated Hospital of Jinan University, Guangzhou, Guangdong, China; Guangdong Provincial Key Laboratory of Traditional Chinese Medicine Informatization, Guangzhou, Guangdong, China

**Keywords:** Merkel cell carcinoma, surgical resection, survival analysis, column chart

## Abstract

**Objective:**

To explore the postoperative risk factors of Merkel cell carcinoma patients who have undergone surgical resection, and to construct a survival prognosis column chart.

**Method:**

Patients diagnosed with Merkel cell carcinoma and underwent surgical resection from 2000 to 2019 were selected from the surveillance, epidemiology, and end results database. COX regression analysis was used to screen for independent prognostic factors, and a column chart was constructed. The predictive performance of the column chart was evaluated using consistency index, receiver operating characteristic curve, and calibration curve.

**Results:**

The results of multi-factor COX regression showed that T stage and N stage were independent prognostic factors affecting cancer-specific survival (CSS) in patients after Merkel cell carcinoma resection. Construct a column chart based on the above two factors. The C-index of the column chart in the modeling group is 0.732 [95% CI (0.649, 0.814)], and the area under the curve (AUC) for the first and second years are 0.816 [95% CI (0.728, 0.904)] and 0.693 [95% CI (0.593, 0.792)], respectively. The C-index in the validation group was 0.724 [95% CI (0.569, 0.879)], and the AUC in the first and second years were 0.739 [95% CI (0.644, 0.833)] and 0.658 [95% CI (0.556, 0.759)], respectively.

**Conclusion:**

The predictive model constructed based on two factors, T stage and N stage, has good prognostic diagnostic accuracy and is helpful for clinical decision-making and personalized treatment.

## 1 Introduction

Merkel cell carcinoma represents a rare yet aggressive form of skin cancer arising from Merkel cells within the skin [1]. Characterized by painless, swiftly expanding patches or nodules, this malignancy frequently manifests on exposed regions like the head, neck, and hands [2]. Diagnosis of Merkel cell carcinoma typically involves live tissue analysis, encompassing biopsy and histopathological examination. Globally, the incidence of Merkel cell carcinoma is relatively low, predominantly affecting elderly individuals, notably Caucasians aged over 50 years, with the risk escalating with advancing age [3,4].

Merkel cell carcinoma, a rare yet aggressive skin neuroendocrine tumor, has garnered significant interest concerning its treatment and prognosis [3]. Postoperative prognosis prediction plays a crucial role in clinical decision-making; however, the research on postoperative risk factors and prognosis prediction for Merkel cell carcinoma patients still lacks depth and comprehensiveness. Previous studies have investigated various postoperative risk factors and prognosis prediction elements, including tumor size, lymph node involvement, and patient age [4–6]. However, these studies often present limitations such as small sample sizes, incomplete study designs, and inadequate data sources. Furthermore, the current literature fails to provide personalized prognosis prediction models for Merkel cell carcinoma patients, impeding the accurate assessment of patient risk and the development of tailored treatment plans by healthcare providers. The objective is to construct a tumor-specific survival analysis-based prognosis prediction model and translate it into an intuitive and practical nomogram chart. By conducting an in-depth analysis of the impact of different postoperative risk factors on patient prognosis, the study aims to enhance the accuracy of assessing patients’ survival risks and offers a more robust foundation for clinical decision-making.

Given the rarity and aggressive nature of Merkel cell carcinoma, treatment plans must comprehensively assess various factors, including patient age, health status, tumor characteristics, and treatment modalities such as surgery, radiotherapy, chemotherapy, and targeted therapy [5,6]. Surgical resection plays a vital role in extending patient survival, although long-term prognosis remains suboptimal [7]. Similar to other types of cancer, accurate individual prognosis prediction is essential for postoperative Merkel cell carcinoma patients. Hence, it is imperative to establish tailored prognostic models for evaluating the postoperative outcomes of Merkel cell carcinoma patients.

The nomogram serves as a dependable and convenient statistical model capable of conducting a comprehensive analysis of all risk factors, extensively applied in forecasting survival outcomes for cancer patients [8,9]. While studies exist on Merkel cell carcinoma postoperative risk prediction line graphs, limitations arise from small sample sizes, resulting in less accurate predictions across various demographic groups. This research project focused on constructing a unique nomogram graph utilizing data from the surveillance, epidemiology, and end results (SEER) database to project cancer-specific survival (CSS) in Merkel cell carcinoma patients’ post-resection, offering clinicians a quantitative instrument for evaluating patient prognoses.

## 2 Materials and methods

### 2.1 Participants

To investigate patients with Merkel cell carcinoma who underwent surgical resection between 2000 and 2019, data extraction was conducted using SEER*Stat software (version 8.4.3) from the SEER database. Inclusion criteria encompassed age range of 18–80 years, pathologically confirmed Merkel cell carcinoma, absence of distant metastasis or prior malignant tumor history, and treatment via surgical resection. Exclusion criteria excluded cases with incomplete clinical data or those that resulted in death within 1 month post-surgery. This research adheres to the Helsinki Declaration guidelines, and due to the public accessibility of the SEER database as a clinical data source, ethical review was deemed unnecessary [10].

### 2.2 Inclusive variables and outcome indicators

The variables included in the study comprise age, gender, race, marital status, radiation, M-stage, T-stage, N-stage, and survival data. The primary outcome of interest is CSS, which denotes the period from the initial diagnosis of Merkel cell carcinoma to either disease-related mortality or the most recent follow-up. To streamline the model-building process, continuous variables are transformed into categorical ones.

### 2.3 Building and validating models

The study population was randomly allocated into a modeling group and a validation group in a 7:3 ratio. The modeling group underwent single-factor COX regression to compute the hazard ratio (HR) along with its 95% confidence interval (CI). Variables with *P* < 0.1 were considered for inclusion in the multiple-factor COX regression analysis to identify the ultimate independent risk factors. Subsequently, a nomogram was developed to forecast the CSS of patients at 1 and 2 years using the outcomes of the multiple-factor COX regression analysis. The predictive accuracy of the nomogram was assessed through various metrics, such as the concordance index (C-index), receiver operating characteristic (ROC) curve, and calibration curve. Lastly, risk scores for all patients were determined based on the nomogram.

### 2.4 Statistical analysis

Continuous variables are summarized by the median and interquartile range, while categorical variables are expressed as frequencies and percentages. The comparison of categorical variables between two patient groups was conducted using the chi-square test or Fisher’s exact test for baseline characteristics. Continuous variables were assessed using the Mann–Whitney *U*-test. Statistical analyses were carried out using R language (version 4.3.2), utilizing key packages such as “readr,” “survival,” “foreign,” “rms,” and “survivalROC.” A two-sided *P*-value below 0.05 was deemed statistically significant in all tests.

## 3 Results

### 3.1 General clinical data

This study included a total of 211 patient data, including 151 in the modeling group and 60 in the validation group. The general clinical data of the included patients are shown in Table 1.

**Table 1:** General clinical data of participants (*n* [%])

Variables	Modeling group (*n* = 151)		Validation group (*n* = 60)		Chi square value	*P*
	*n*	%	*n*	%		
**Age (years)**						
50–64.9	31	20.5	10	16.7	2.039	0.361
65–79.9	84	55.7	30	50.0		
80–	36	23.8	20	33.3		
**Sex**						
Female	58	38.4	15	25.0	2.846	0.092
Male	93	61.6	45	75.0		
**Race**						
White	146	96.7	59	98.3	0.358	0.849
Black and other	5	3.3	1	1.7		
**Marital**						
Unmarried	52	34.4	21	35.0	0.006	0.938
Married	99	65.6	39	65.0		
**Radiation**						
No	50	33.1	22	36.7	0.109	0.741
Yes	101	66.9	38	63.3		
**M stage**						
M0	145	96.0	59	98.3	0.175	0.676
M1	6	4.0	1	1.7		
**N stage**						
N0	81	53.6	31	51.7	5.398	0.145
N1	67	44.4	24	40.0		
N2 and N3	2	1.3	2	3.3		
NX	1	0.7	3	5.0		
**T stage**						
T1	82	54.3	29	48.3	0.533	0.137
T2	31	20.5	7	11.7		
T3 and T4	8	5.3	6	10.0		
TX	30	19.9	18	30.0		
**Survival time (months)**						
1–18	70	46.4	23	38.3	0.819	0.365
19–	81	53.6	37	61.7		
**Status**						
Alive	126	83.4	47	78.3	0.453	0.501
Dead	25	16.6	13	21.7		

### 3.2 Prognostic factor analysis

Univariate Cox regression analysis results indicated that prognostic factors for Merkel cell carcinoma patients post-surgery encompass age, M-stage, T-stage, and N-stage (*P* < 0.05), as presented in Table 2. Multivariable Cox regression analysis employing the Enter method identified T-stage and N-stage as autonomous prognostic contributors to patient survival.

**Table 2:** Results of COX regression analysis for single and multiple factors in the modeling group

Variables	Univariate analysis		Multivariate analysis	
	HR (95%CI)	*P*	HR (95%CI)	*P*
**Age (years)**				
50–64.9	Ref.		Ref.	
65–79.9	1.46 (0.41,5.18)	0.558	1.45 (0.39,5.40)	0.575
80–	3.33 (0.91,12.10)	0.068	3.25 (0.87,12.18)	0.08
**Sex**				
Female	Ref.		—	—
Male	1.49 (0.64,3.46)	0.355	—	—
**Race**				
White	Ref.		—	—
Black and others	2.89 (0.68,12.32)	0.151	—	—
**Marital**				
Unmarried	Ref.		—	—
Married	0.94 (0.42,2.13)	0.881	—	—
**Radiation**				
No	Ref.		—	—
Yes	0.91 (0.40,2.06)	0.818	—	—
**M stage**				
M0	Ref.		Ref.	
M1	4.19 (1.25,14.02)	0.020	1.39 (0.31,6.31)	0.666
**N stage**				
N0	Ref.		Ref.	
N1 and others	3.35 (1.40,8.04)	0.007	3.84 (1.46,10.11)	0.006
**T stage**				
T1	Ref.		Ref.	
T2	1.04 (0.33,3.26)	0.949	0.59 (0.18,1.92)	0.379
T3 and T4	4.00 (1.27,12.58)	0.018	4.23 (1.04,17.15)	0.043
TX	1.86 (0.68,5.05)	0.224	0.98 (0.34,2.83)	0.971

### 3.3 Establishment and verification of survival nomogram

**Figure 1:**
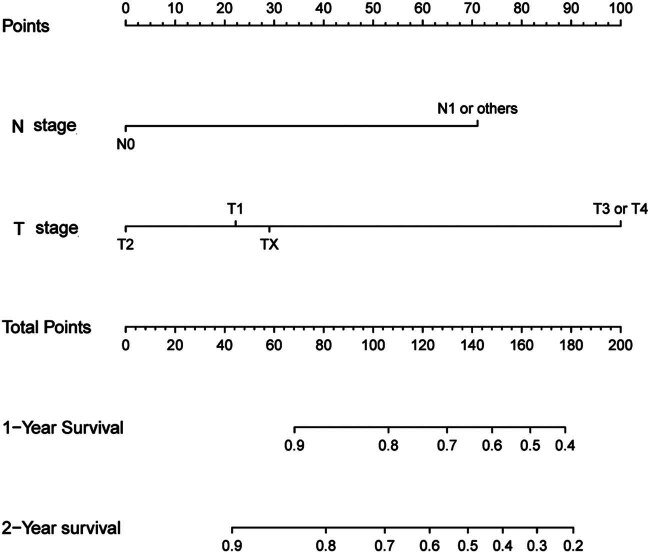
Prediction of survival nomogram in Merkel cell carcinoma patients after surgical resection.

**Figure 2:**
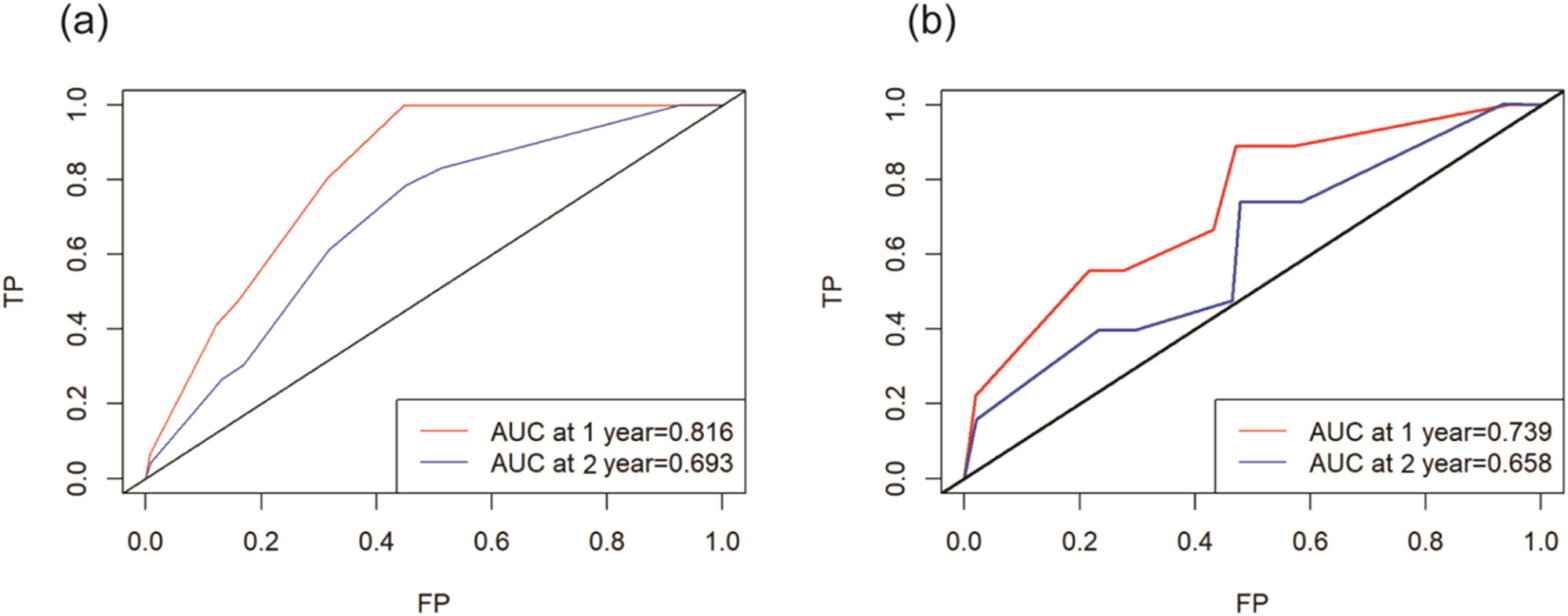
ROC curves of modeling and validation groups: (a) modeling group and (b) validation group.

**Figure 3:**
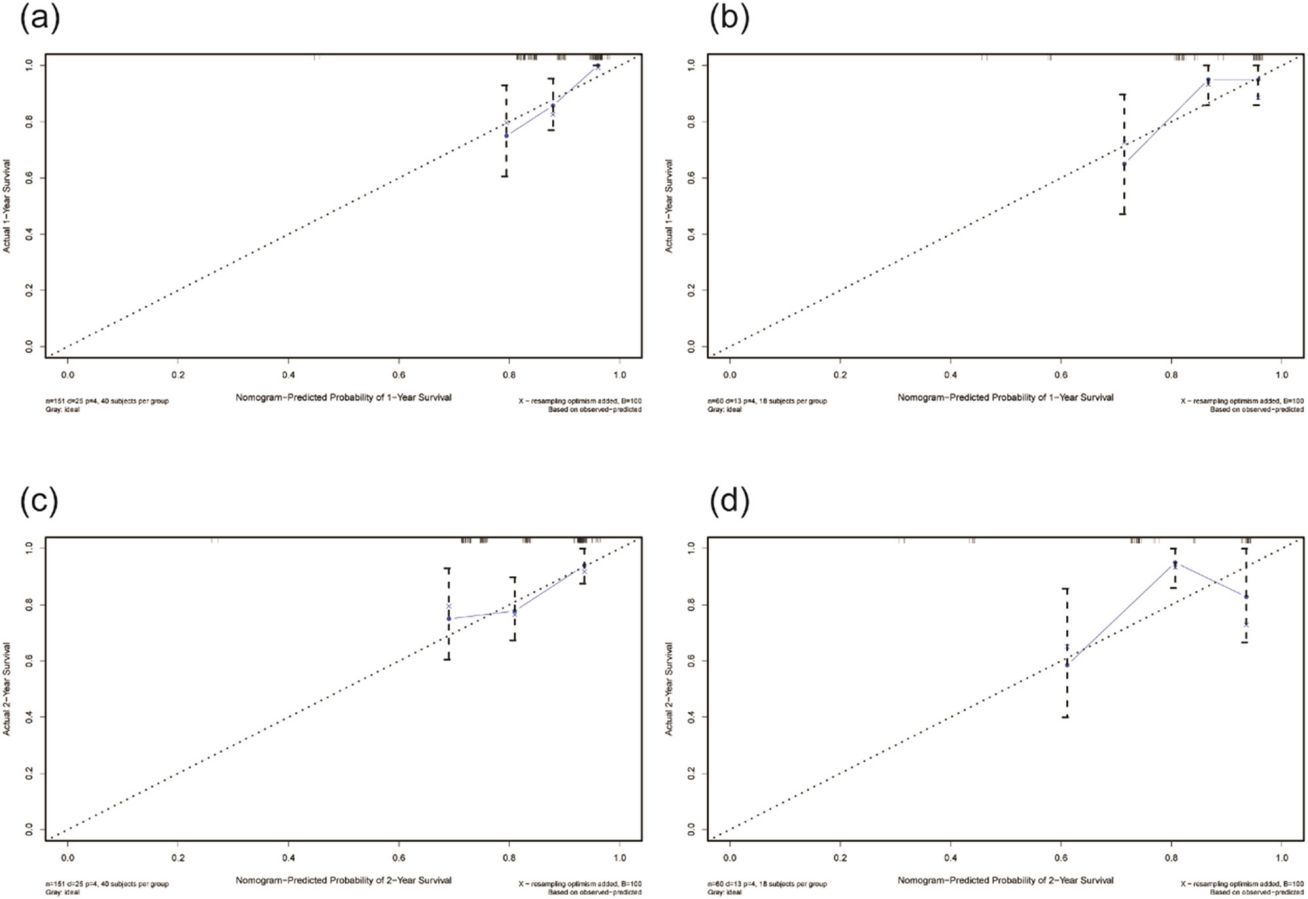
Calibration curves of modeling and validation groups: (a) calibration curve of the modeling group for 1 year, (b) calibration curve of the modeling group for 2 years, (c) calibration curve of the validation group for 1 year, and (d) calibration curve of the validation group for 2 years.

The results of the multivariable Cox regression analysis led to the development of a survival nomogram (Figure 1). In the modeling group, the nomogram estimated the CSS with a C-index of 0.732 [95% CI (0.649, 0.814)], while in the validation group, the C-index was 0.724 [95% CI (0.569, 0.879)], indicating high prognostic accuracy of the model. The ROC curve illustrated that the predicted area under the curve for CSS in the first and second years were 0.816 [95% CI (0.728, 0.904)] and 0.693 [95% CI (0.593, 0.792)] in the modeling group, and 0.739[95% CI (0.644, 0.833)] and 0.658 [95% CI (0.556, 0.759)] in the validation group (Figure 2). Calibration curves for both modeling and validation groups closely resembled the diagonal line for the first and second years, demonstrating strong alignment between the model’s predictions and actual data. Overall, this model exhibits effective performance in forecasting the prognosis of patients following Merkel cell carcinoma surgery (Figure 3).

### 3.4 Construct survival curve based on nomogram

**Figure 4:**
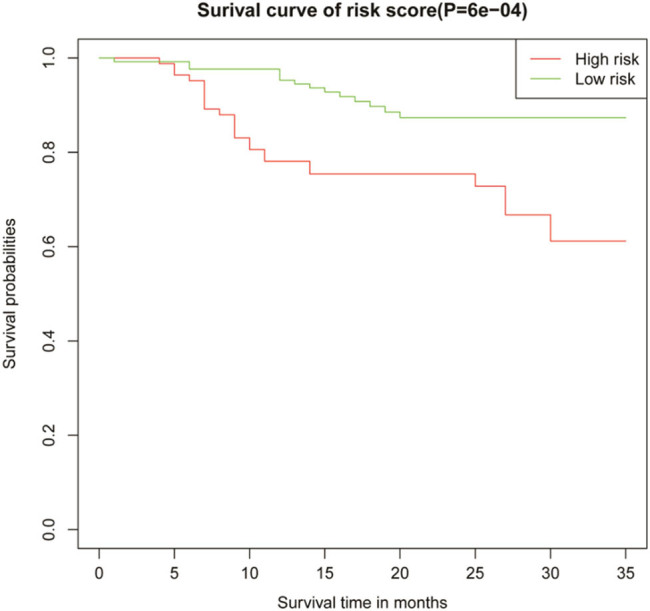
Survival curves of Merkel cell carcinoma patients with different risk groups after surgical resection.

According to the median of patient risk scores, they were divided into low-risk and high-risk groups (high-risk group were higher than median of patient risk scores, and low-risk group were lower than median of patient risk scores), and the CSS between the two risk groups was compared using Kaplan–Meier analysis and log-rank testing. As shown in Figure 4, the difference in CSS between the two risk subgroups was statistically significant (*P* < 0.001).

## 4 Discussion

Merkel cell carcinoma is recognized as a highly aggressive tumor with a poor prognosis. Despite its rarity among skin cancers, Merkel cell carcinoma’s aggressive behavior and rapid growth make it prone to infiltrating surrounding tissues and other regions, resulting in delayed detection and unfavorable outcomes [11,12]. Research indicates a 5-year survival rate for Merkel cell carcinoma ranging from 30 to 77% [13,14]. Given its highly invasive nature and propensity for metastasis, timely treatment and meticulous follow-up are essential for patient survival [15]. Treatment strategies typically encompass a multidisciplinary approach involving surgical resection, radiation therapy, chemotherapy, and targeted therapies to effectively manage tumor progression and minimize the risk of recurrence and metastasis [16]. Therefore, early detection, prompt intervention, and continuous monitoring play a pivotal role in enhancing the survival and prognosis of Merkel cell carcinoma patients. Patients and healthcare professionals should vigilantly track disease progression and implement appropriate therapeutic and management strategies to enhance patient outcomes and quality of life [17]. This study utilized data from the SEER database to construct a nomogram for predicting CSS following Merkel cell carcinoma resection, conducting internal validation, and thoroughly assessing the nomogram’s predictive accuracy and efficacy.

The N stage refers to the evaluation of lymph node metastasis in patients with Merkel cell carcinoma [18]. The findings of this research indicated that staging at N1 or higher serve as an independent prognostic factor for patients undergoing surgical resection of Merkel cell carcinoma. Lymph node metastasis is primarily disseminated through the lymphatic system in Merkel cell carcinoma, and its presence often signifies tumor progression and deterioration, predisposing patients with lymph node involvement to increased risks of recurrence and mortality [19]. Assessing the N stage is crucial in determining the necessity for additional interventions like lymph node dissection or radiation therapy. The detection of lymph node metastasis may require modifications to a patient’s treatment plan to optimize treatment efficacy and prognosis [20]. By utilizing the N stage assessment, healthcare providers can conduct more precise prognostic evaluations and propose tailored treatment strategies. Prognostic determinations facilitate the collaborative formulation of post-treatment strategies and the vigilant monitoring of patient follow-up and management. Through a comprehensive evaluation of N stage results, physicians can enhance treatment planning and implement more efficient treatment and management approaches to advance patient survival rates and enhance quality of life.

The T stage involves the categorization of tumors based on clinical and pathological data to aid healthcare professionals in assessing the severity and prognosis of the tumor. Utilizing the T stage post-surgical resection can enhance physicians’ ability to predict the patient’s prognosis, particularly in cases of Merkel cell carcinoma [21]. This research identified T3 and T4 stages as autonomous prognostic indicators for individuals receiving surgical treatment for Merkel cell carcinoma. Assessment of the T stage is predominantly dependent on the tumor’s size and level of invasiveness, typically categorized into four stages ranging from T1 to T4, where a higher T value signifies a larger or more aggressive tumor. Specifically for Merkel cell carcinoma patients, the T stage serves as a direct indicator of tumor growth and dissemination, thus aiding in prognosis prediction [22]. Generally, an elevated T stage indicates a larger and more aggressive tumor, potentially leading to a less favorable prognosis for the patient. Therefore, the T stage plays a crucial role as an independent prognostic marker in forecasting outcomes for patients undergoing surgical resection for Merkel cell carcinoma.

This study presents the findings of a tumor-specific survival analysis conducted on patients with Merkel cell carcinoma using data from the SEER database, offering valuable insights for clinical practice. The results indicate a close association between certain postoperative risk factors, such as tumor size and lymph node involvement, and patients’ survival risk. These results underscore the importance of enhanced monitoring and personalized treatment plans for postoperative patients to improve survival rates and mitigate the risk of recurrence. In contrast to previous research studies, this study boasts a larger sample size and enhanced research methodology, thereby bolstering the reliability and generalizability of the outcomes [6,19,22]. These findings align with existing literature, further affirming the correlation between specific risk factors and the prognosis of Merkel cell carcinoma patients. In clinical settings, the study’s conclusions can assist healthcare providers in accurately evaluating the survival risk of postoperative Merkel cell carcinoma patients and formulating tailored follow-up and treatment strategies. Furthermore, through comparative analysis with other research findings, the study validates its conclusions, establishing a robust foundation for the clinical implementation of tumor-specific survival analysis. Future investigations should focus on enlarging the sample size and incorporating comprehensive clinical data to further validate the study’s outcomes and provide conclusive evidence in support of personalized treatment and clinical management of Merkel cell carcinoma patients.

The study has several limitations. First, relevant factors affecting the prognosis of patients’ post-surgical resection of Merkel cell carcinoma, such as surgical margins and serum tumor markers, were not accessible in the SEER database and thus were excluded from the analysis. Moreover, due to its retrospective nature, this research is restricted by the constraints of the SEER database, posing challenges in updating to the most current data and possibly leading to selection bias within the study cohort. Additionally, the absence of external multi-institutional validation data in this study limits the broader applicability of its findings.

## 5 Conclusion

T and N stages, as autonomous prognostic indicators of tumors, directly reflect the tumor’s size, invasiveness, and lymph node involvement, crucial factors strongly associated with patient survival. Higher T and N stages correlate with a poorer prognosis, necessitating more assertive treatment and vigilant monitoring. This study’s findings underscore that N and T staging serve as independent risk factors influencing the outcomes of patients undergoing surgical resection for Merkel cell carcinoma. Through a comprehensive evaluation of these factors, clinical practices can be more effectively guided, positively influencing patient recovery and long-term survival.
